# BactoSpin: Novel Technology for Rapid Bacteria Detection and Antibiotic Susceptibility Testing

**DOI:** 10.3390/s21175902

**Published:** 2021-09-02

**Authors:** Vlad Shumeiko, Guy Hidas, Chen Nowogrodski, Yariv Pinto, Ofer Gofrit, Mordechai Duvdevani, Oded Shoseyov

**Affiliations:** 1 Faculty of Agriculture, Food and Environment, The Hebrew University of Jerusalem, Rehovot 7610001, Israel; vlad.shumeiko@mail.huji.ac.il (V.S.); Chen.Nowo@mail.huji.ac.il (C.N.); 2 Department of Urology, Hadassah Medical Center, Faculty of Medicine, Hebrew University of Jerusalem, Jerusalem 9112001, Israel; Guyh@hadassah.org.il (G.H.); roferg@hadassah.org.il (O.G.); Duvdevam@hadassah.org.il (M.D.); 3 Center for Functional and 3D Printing, Center for Nanoscience and Nanotechnology, The Hebrew University of Jerusalem, Jerusalem 9190401, Israel; yariv.pinto@mail.huji.ac.il

**Keywords:** AST, UTI, microfluidics

## Abstract

Inappropriate use of antibiotics is one of the leading causes of the increasing numbers of resistant bacteria strains, resulting in 700,000 deaths worldwide each year. Reducing unnecessary use of antibiotics and choosing the most effective antibiotics instead of broad-spectrum drugs will slow the arms race between germs and humans. Urinary tract infections (UTIs) are among the most common bacterial infections. Currently, accurate diagnosis of UTI requires approximately 48 h from the time of urine sample collection until antibiotic susceptibility test (AST) results. This work presents a rapid bacterial detection device that integrates a centrifuge, microscope, and incubator. Two disposable microfluidic chips were developed. The first chip was designed for bacteria concentration, detection, and medium exchange. A second multi-channel chip was developed for AST. This chip contains superhydrophobic and hydrophilic coatings to ensure liquid separation between the channels without the need for valves. The designed chips supported the detection of *E. coli* at a concentration as low as 5 × 10^3^ cells/mL within 5 min and AST in under 2 h. AST was also successfully performed with *Klebsiella pneumonia* isolated from a human urine sample. In addition, machine-learning-based image recognition was shown to reduce the required time for AST and to provide results within 1 h for *E. coli* cells. Thus, the BactoSpin device can serve as an efficient and rapid platform for UTI diagnostics and AST.

## 1. Introduction

According to the Centers for Disease Control and Prevention (CDC), more than 2.8 million antibiotic-resistant infections occur in the U.S. each year, and more than 35,000 people die as a result [1]. In addition to clinical burdens, approximately USD 3 trillion in annual GDP is lost due to excess health care and loss of productivity associated with antimicrobial resistance [2,3]. Inappropriate use of antibiotics is one of the leading causes of the increasing numbers of resistant bacteria strains [4]. A strong relationship was documented between country-wide antibiotic consumption and the prevalence of nonsusceptible bacteria [5]. Urinary tract infections (UTIs) are among the most common bacterial infections, affecting more than 150 million people worldwide annually [6]. Those who suffer from recurrent infections can have more than three cases of UTI per year [7]. Women are more prone to UTI, with more than 50% of women experiencing at least one UTI in their lifetime [8]. *E. coli* is the most common cause of community-acquired UTI and is responsible for more than 80% of the cases [9].

UTI diagnostics involves multiple steps, integrating various techniques and equipment. Usually, after the initial patient examination, a dipstick urinalysis is performed, which has a sensitivity of only around 70% [10,11]. Microscopy analysis is a possible alternative, reportedly increasing the sensitivity to more than 92% [12,13], but is less standardized, more expensive, time consuming, and still not sufficiently accurate. In the majority of clinical microbiology laboratories, the next step in the UTI diagnostics process relies on bacterial culture [14]. Usually, bacterial culture requires 18–48 h and is performed in centralized clinical laboratories, requiring sample handling and shipping [15]. After the 18–48 h incubation, colony-forming units (CFUs) are counted. Up to 70% of urine culture results test negative at this stage, resulting in time and money spent on unnecessary testing [16]. Next, the bacterial cells are subjected to an identification process, which can be performed using a number of methods, including chromogenic media, mass spectrometry, and various biochemical methods that are often performed with semiautomatic platforms, e.g., Vitek2 (bioMérieux, Marcy-l’Étoile, France), Phoenix (Becton-Dickenson, Franklin Lakes, New Jersey, United States), and MicroScan Walkaway (Siemens, Munich, Germany) [17]. The next and the last step is antibiotic susceptibility testing (AST), which requires an additional 18–24 h. AST is performed using a disc susceptibility method, broth microdilution, or antibiotic strips (E-test). The procedure is performed manually or using the semiautomatic platforms mentioned above.

Overall, it can take more than 72 h from sample collection until AST results are available. Due to this long delay in diagnostics, physicians frequently initiate broad-spectrum antibiotics, which significantly contributes to the emergence of resistant pathogens, chronic infections, ineffective treatment, and increased care costs [18]. Moreover, while there has been progress in the development of new systems for AST [4,19], and many of the proposed technologies are accurate and rapid, they are often costly. The cost of the standard diagnostic process, from the urine sample to AST results, including identification, is approximately USD 10 (20). Thus, a novel methodology is needed for rapid and accurate bacteria detection and AST at a competitive price.

Microfluidics is an emerging technique for point-of-care (POC) diagnostics. Diagnostic microfluidic devices have evolved rapidly from early single-channel structures to complex systems that can simultaneously perform hundreds of tests [20]. In the field of microfluidics, movement of the liquid from one part of the chip to another can be achieved using various techniques, including syringe and peristaltic pumps, electrochemical bubble generation, acoustics, magnetics, electrokinetics, and centrifuge [21]. The centrifugal-based microfluidic platforms offer a number of advantages compared to other techniques. Centrifugal pumping requires less instrumentation, involving only a compact and simple motor to create the centrifugal force [22]. It also does not require any external tubing connection to drive the liquid, which reduces the risk of contamination. If the assay involves powders that must be dissolved in the liquid sample, the same centrifuge motor can be used to shake the chip, to enhance the process. Finally, when the analysis involves a microscope, the rotor can be rotated to enable examination of different fields in the chip, with no need to move the microscope objective itself. 

Besides liquid pumping, valves that prevent unwanted liquid mixes are one of the essential components in microfluidic devices. Capillary valves [23], including hydrophobic and hydrophilic parts, are an exciting alternative to classical valves as they involve no moving parts and are much simpler to manufacture.

Centrifuge-based microfluidics may enable the acceleration of each of the steps of bacterial infection diagnostics, eliminating the need for multiple devices and enabling on-site diagnostics, thus significantly reducing diagnostic time and potentially also the overall costs. The current work presents a centrifuge-driven microfluidic chip that can semiautomatically perform the essential steps for UTI diagnostics, i.e., bacteria concentration, isolation, purification, and AST. 

## 2. Materials and Methods

### 2.1. Chemicals and Reagents

Ampicillin, gentamicin, amikacin and Lysogeny broth were ordered from Tivan Biotech, Kfar-Saba, Israel. 1H,1H,2H,2H-perfluorooctyltriethoxysilane (FOTS), 97%, was purchased from Alfa Aesar, Thermo Fisher Scientific, Waltham, MA, USA, and P 25 TiO_2_ (718467) was purchased from Sigma Aldrich, Rehovot, Israel. Absolute ethanol was purchased from Romical, Israel. PETG and PLA were ordered from 4project (Israel). VeroClear is produced by Stratasys. CF-343 silicon glue (Hercules) was purchased from a local shop. 

### 2.2. Chip Design and Production

**Figure 1:**
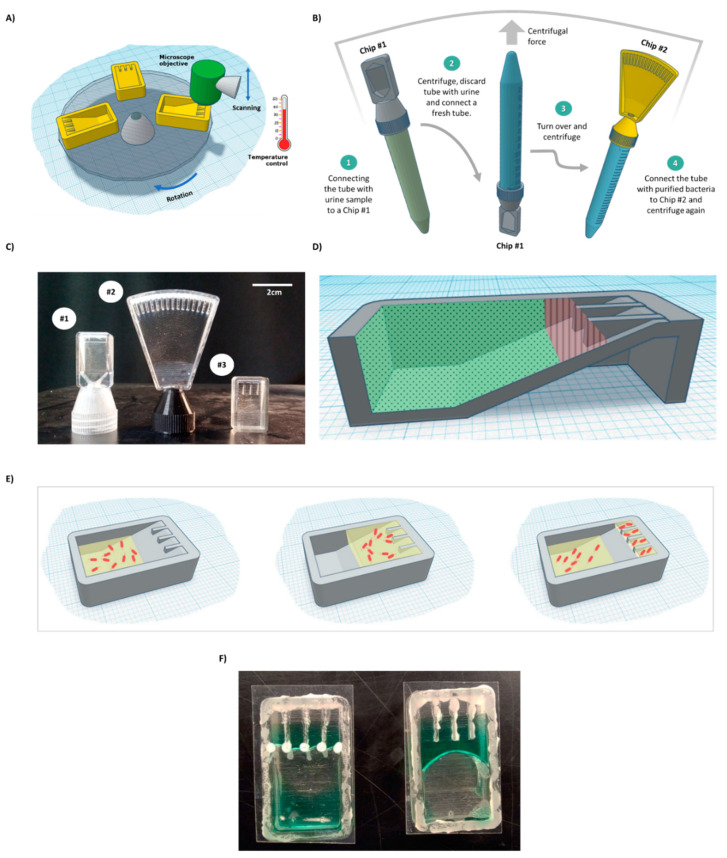
BactoSpin system. (**A**) Scheme of the proposed device that combines a centrifuge to push the cells into the channels, a microscope to assess bacterial cell morphology and growth, and an incubator to maintain optimal temperature for bacteria growth. (**B**) A scheme of the proposed UTI diagnosis process, medium exchange, and AST using the BactoSpin system. (**C**) A photo of 3D-printed BactoSpin chips for UTI diagnostics and AST. Chip #1 was designed to concentrate bacteria from urine samples into a single chamber for UTI diagnostics, cells count and medium exchange. Chip #2 is a 16-channel version of the chip designed to perform the AST and Chip #3 is a 4-channel version. (**D**) Schematic presentation of areas coated with a hydrophilic (green dots) and with a superhydrophobic (red lines) coating. (**E**) During centrifugation, medium with the bacteria cells is forced into the channels. After centrifugation stops and due to applied coatings, the excess liquid flows back, enabling separation between the channels. (**F**) A photo of the chip with superhydrophobic and hydrophilic coatings (left) and without coatings (right).

Three microfluidic chips (Chip #1, Chip #2, and Chip #3) were designed using Autodesk Tinkercad and SolidWorks software (Figure 1C). Chip #1 has a single channel and is used for bacteria detection. Chip #2 and Chip #3 are multi-channel chips for the AST. For initial prototyping, the 3D chip model was sliced using Ultimaker Cura 4.5 software and printed with an Ender printer using a white PLA filament. A centrifuge adapter (Supplemental Materials; Figure S1) and microscope adapter (Supplemental Materials; Figure S2). were printed with an Ender printer, using PETG and PLA, respectively. For experiments involving bacteria cells, the 3D chip model was sliced with GrabCAD and printed with a Stratasys J826 Polyjet printer using VeroClear photopolymer and SUP 706B support. The chip was sonicated for 15 min in 100% ethanol before use.

The superhydrophobic coating for the chip was prepared as described elsewhere, with minor modifications [24]. Briefly, 1% *w*/*w* FOTS was mixed with pure ethanol, and the solution was vortexed for 5 min at room temperature. P 25 TiO_2_ nanoparticles (13% *w*/*w*) were added, and the solution was vortexed for an additional 10 min. The superhydrophobic coating was applied on the chip with a 10 uL Hamilton glass syringe. 

The volume of a single channel in Chip #3 used for the AST was 28 uL. Antibiotics were drop-dried into the channels at breakpoint concentration, as indicated by the FDA [25]—8 µg/mL for ampicillin, 4 µg/mL for gentamicin, and 16 µg/mL for amikacin.

Glass or transparent polystyrene sheets were used to close the chip (cap). CF-343 silicon glue (Hercules) was applied using a Hyrel3D HYDRA printer with a 1 mL syringe and a 21G needle to bind and seal between the cap and the chip (Supplemental Materials, Figure S1 and Video S1). The glue was left to dry for 24 h before the chip was used.

### 2.3. Bacteria Detection in 10 mL Sample

*E. coli* cells were grown overnight in lysogeny broth (LB). Bacterial cell culture density was calculated by OD600 of 1.0 = 8 × 10^8^ cells/mL and samples were diluted to the desired densities. To imitate UTI detection, *E. coli* cells were spiked into urine obtained from a healthy volunteer (10 mL of 5 × 10^3^ cells/mL). Urine was injected into a standard 15 mL tube, which was connected to Chip #1, directly inserted upside-down into a benchtop centrifuge (Eppendorf centrifuge 5910 R) without any adapter, and centrifuged for 8 min, at 1900 rpm. Excess liquid from the chip was returned to the 15 mL tube by manually rotating the tube (Supplement files, Video S2). Concentrated cells were observed using an EVOS FL Auto microscope with a 60× objective.

### 2.4. Monitoring Bacterial Reaction to Antibiotics in the Chip

*E. coli* cells in LB medium (300 uL of 5 × 10^5^ cells/mL) were injected directly into Chip #3, briefly centrifuged for 10 s at 800 rpm (Sigma 1–15 centrifuge with 3D-printed adapter: Supplemental materials, Figure S1) to push the liquid into the channels, and then incubated for different durations of time, at 37 °C. Photos of the channels were taken, at various magnifications, using an EVOS FL Auto microscope and a custom adapter (Supplemental Materials, Figure S2).

### 2.5. Machine Learning for AST

*E. coli* cells (5 × 10^5^ cells/mL) were incubated in 24-well plates (Corning**^®^**) and photographed every 30 min, with an EVOS FL Auto microscope, using a 20× objective. Each photo was split into 100 images using Python. Some wells were randomly selected to serve as a training set, while others served as a test set. Overall, approximately 750 images were used for training and around 350 for the test. For machine learning (ML), an open-source Python library ImageAI and ResNet algorithms were used [26]. 

### 2.6. Human Urine Sample

A human urine sample collected from a volunteer UTI patient was obtained from Hadassah Medical Center (Jerusalem, Israel). The experiment was approved by the Ethics Committee of Hadassah University Hospital (IRB number 04-2021). The sample was transferred on ice to the laboratory and was passed through a 5-micron PES syringe filter. Next, the sample was introduced to an LB agar petri dish and incubated overnight under 37 °C. A single colony was selected and incubated for an additional 12 h in liquid LB. Bacteria were then diluted to a concentration of 5 × 10^5^ bacteria cells/mL and were injected into Chip #3 containing drop-dried antibiotics. The chip was mounted into the centrifuge using a customized adapter and briefly centrifuged for 10 s, at 800 rpm, as described above. The chip was then transferred to the incubator and incubated for 2 h and for 4 h before being imaged under the EVOS FL Auto microscope.

## 3. Results and Discussion

### 3.1. System Design 

This work aimed to develop a system that can perform most of the steps necessary for UTI diagnostics and AST in a single machine. As mentioned above, currently, most clinical laboratories rely on an overnight culture of urine samples to obtain a definitive indication of the presence of bacteria and CFU count. There are no consensus criteria regarding the minimal CFU defining UTI, and it varies greatly across laboratories, with 10^3^ CFU being the smallest reported count used to define UTI [27]. After obtaining a count, bacteria are purified from the urine—as urine may have factors that will interfere with the AST, for example, in the case when the patient is already taking an antibiotic treatment. In the next step, the concentration of bacteria cells must be adjusted to that required for the AST assay (5 × 10^5^ cells/mL). The next and final step is AST itself.

The current work presents the concept of a device (Figure 1A–E) that combines a microscope, a centrifuge, an incubator, and microfluidic chips for the semiautomatic processing of urine samples suspected to be carrying infectious bacteria. 

**Figure 2:**
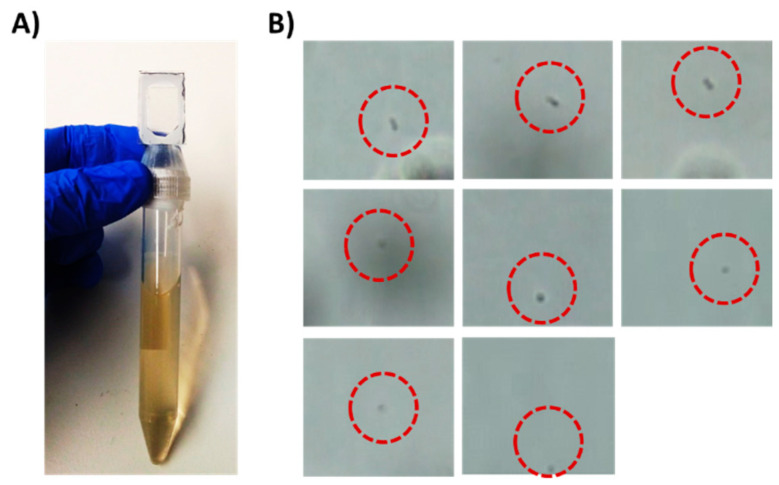
Detection of 5 × 10^3^ E. coli cells/mL in a liquid sample as a model for UTI diagnostics. (**A**) A 15 mL tube with Chip #1 attached to it. The tube holds 10 mL sample spiked with E. coli cells. (**B**) Trapped bacterial cells as observed under a brightfield microscope equipped with 60× objective after centrifugation.

Chip #1 (Figure 1C) was used for bacteria detection and was designed with an adaptor, which acts as a cap for standard 15 mL tubes. On the top side of the chip, a single triangular channel, 50 microns in height, was used for cell concentration and observation. *E. coli* cells (5 × 10^3^) were spiked into 10 mL human urine (prefiltered through a 5-micron syringe filter) obtained from a healthy volunteer in a 15 mL tube, which was then inserted into a centrifuge with the chip pointing down. The sample was centrifuged for 8 min. The chip was designed to enable easy flow of the medium to the end of the chip during centrifugation and easy flow back to the tube when the tube is taken out of the centrifuge (Supplemental Files; Video S2). The concentrated bacteria became trapped in the channel, as observed under a microscope (Figure 2). This step enabled easy bacteria detection in under 10 min and effectively concentrated samples with as few as 5 × 10^3^ cells/mL. While in the current work, the concentrated cells were observed and manually detected, for the future, it has been proposed to use image recognition software that will enable automatic bacteria detection and counting.

After initial detection, bacterial cells must be transferred from urine to a suitable medium for further growth. This can be achieved by connecting a fresh tube to the chip and inverting the complex, such that the chip points upward (Figure 1B, Steps 2 and 3). Brief centrifugation should bring the bacterial cells trapped in the chamber down to the new tube. Fresh dedicated growth medium can be added to the tube before centrifugation. After centrifugation, the final concentration can be adjusted as needed. Then, the tube with the bacteria is connected to Chip #2 for the AST assay (Figure 1B, Step 4) and briefly centrifuged to bring the medium with the cells to the channels of Chip #2 containing dried antibiotics. In the current work, due to extensive leakage from the 3D-printed prototype (caused by a poor fit of the chip to the thread on a standard 15 mL tube) and safety reasons, 5 × 10^5^ bacterial cells in LB were directly injected into a simplified Chip #3 (Figure 1C) to demonstrate AST. 

### 3.2. Designing a Chip for AST

Growing bacterial cells in isolated wells, each containing different antibiotics at specific concentrations, is a well-established system for AST [28,29]. Here, we propose a chip that contains multiple small channels for bacterial cell incubation. We designed Chip #3 with 4 channels and Chip #2 with 16 channels (Figure 1C). One channel in each chip serves as a control, while the others contain drop-dried antibiotics at breaking point concentrations [25]. The sample can be introduced via a 15 mL tube (Figure 1B, Step 4) or injected directly into the chip. Brief centrifugation will force the liquid with the bacterial cells into the channels (Figure 1E); such liquid would otherwise not enter the channels (Supplemental Materials, Figure S3). 

**Figure 3:**
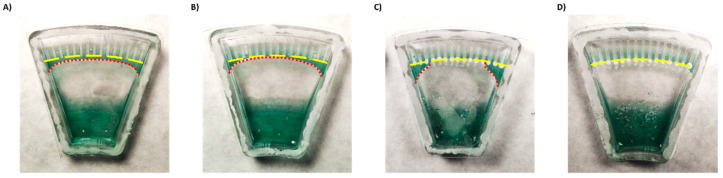
Demonstration of liquid backflow and channel separation after centrifugation of a chip (**A**) without coatings, (**B**) with a hydrophilic coating only, (**C**) with a superhydrophobic coating only, and (**D**) with both hydrophilic and superhydrophobic coatings. Yellow lines indicate channel entrances. Red lines—excess liquid.

To create isolated environments for testing each antibiotic, there must be a separation between channels. For this purpose, the excess liquid must be removed, and capillary flow between the channels must be prevented. If the chip is used as is, the liquid remains stuck in the upper part of the chip (Figure 1F and Figure 3A). However, excess liquid can be passively brought back to the bottom part of the chip after centrifugation stops without physically rotating the chip, by designing regions within the chip with different levels of hydrophobicity. 

First, to enhance backflow of the excess liquid to the main reservoir, we coated the lower part of the chip with a silicone surfactant BYK-348. BYK-348 significantly reduces the water contact angle, thus drawing the liquid back. However, despite some improvement (Figure 3B), part of the liquid still remained and connected between the channels driven by a capillary force (Supplemental Materials Figure S4). 

To try to enhance separation between the channels, the top part of the chip was treated with a superhydrophobic coating. Surface coated with superhydrophobic paint based on titanium FOTS-coated dioxide nanoparticles (with a particle size of 21 nm), a combination which creates a lotus effect [24,30], exhibited a higher water contact angle compared to uncoated surface (Supplemental Materials Figure S5). However, while the coating effectively separated the middle channels, superhydrophobic coating alone was insufficient to fully isolate the side channels (Figure 3C). 

**Figure 4:**
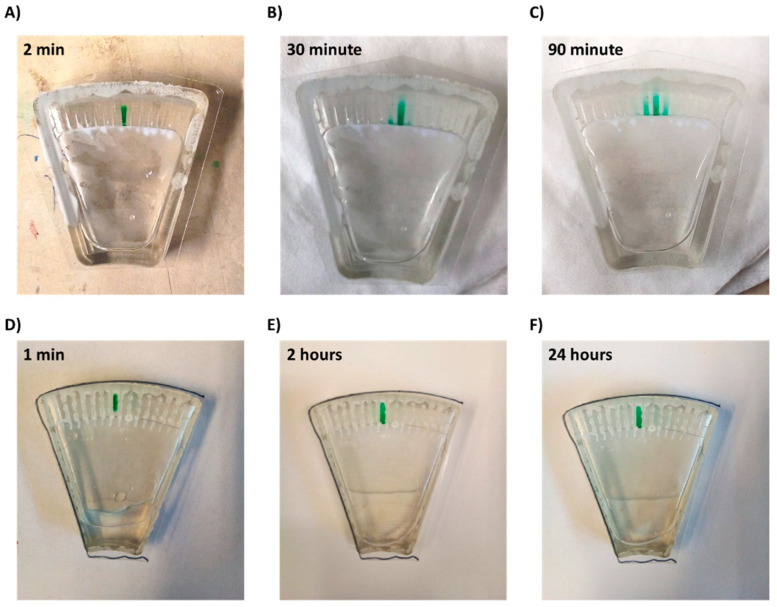
Diffusion of a drop-dried dye from a microchannel within the chip. (**A**–**C**) Chip without superhydrophobic and hydrophilic coatings and (**D**–**F**) chip with coatings.

Thus, a combination of both hydrophilic coating and superhydrophobic coating was applied. This approach enabled full and spontaneous backflow of the medium and effective complete separation between the channels in both Chip #2 and Chip #3 (Figure 1F and Figure 3D; Supplemental materials: Videos S3–S6). A green food color drop-dried in the coated channels remained in the channel, even after a 24 h incubation (Figure 4D–F and Figure S6A), while in the uncoated chip, the color diffused out within less than 30 min (Figure 4A–C and Figure S6B), indicating efficient separation of the channels when the coating is applied. Moreover, these results also propose that dried antibiotics inside the channels will not have enough time to diffuse out of the channels during the short centrifugation stage.

### 3.3. In-Chip AST of E. coli Cells

**Figure 5:**
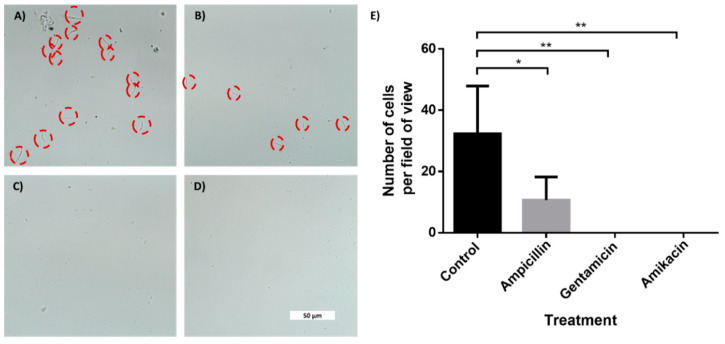
In-chip antibiotic sensitivity testing of an E. coli-spiked sample. Brightfield microscopy photos of E. coli cells taken with 60× objective after 2 h of incubation. The cells were incubated inside microchannels with an entrance coated with a superhydrophobic coating. (**A**) Control channel. Channel with drop-dried (**B**) ampicillin, (**C**) gentamicin and (**D**) amikacin. (**E**) Manual cells count per field of view (690 μm × 300 μm) using a 20× magnification. n = 3. Error bars indicate standard deviation (SD). * *P* ≤ 0.05, ** *P* ≤ 0.01.

To demonstrate AST, we used the four-channel chip with superhydrophobic and hydrophilic coatings. One channel served as a control, while three others held drop-dried ampicillin, gentamicin and amikacin. We designed and printed an adaptor to hold the chip in a standard benchtop centrifuge (Supplemental Files; Figure S7). After coatings, the chip was closed with a glass lid using silicone glue. *E. coli* cells (5 × 10^5^) in LB medium were injected into the chip via a small hole in the back, briefly centrifuged, and were incubated for 2 or 4 h. As shown in Figure 5 and Figure S8, a clear difference in cell counts was observed between the control channel and channels containing drop-dried antibiotics, indicating their susceptibility to tested antibiotics. While after 2 h, the difference was only discernible using high magnification (Figure 5A–D), after 4 h of incubation, the differences in cell numbers were clearly seen even with a low-magnification objective (Supplemental Figure S8). The fact that *E. coli* cells grew extensively in the control channel indicated that the superhydrophobic and hydrophilic coatings prevented the antibiotics from diffusing out of the test channels to the control channel. The antibiotics efficiently diffused inside the test channels, as evidenced by the absence of bacterial growth along the length of the channels that contained antibiotics. 

### 3.4. In-Chip AST of Bacteria Isolated from a Clinical Urine Sample

**Figure 6:**
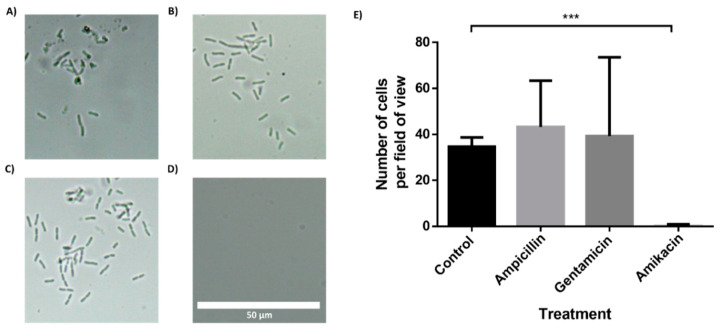
Antibiotic susceptibility testing of Klebsiella pneumoniae, isolated from a clinical urine sample. The multi-channel chip with superhydrophobic and hydrophilic coatings was loaded with a K. pneumonia in LB and incubated for 2 h. (**A**) Control channel. Channel with drop-dried (**B**) ampicillin, (**C**) gentamicin and (**D**) amikacin. (**E**) Manual cell count per field of view (235 μm × 100 μm) using a 60× magnification and a brightfield microscope. n = 3. Error bars indicate standard deviation (SD). *** *P* ≤ 0.001.

To further demonstrate the feasibility of the BactoSpin system, AST was performed using bacteria isolated from a urine sample collected from a UTI patient. The bacteria in the urine sample had been identified as *Klebsiella pneumoniae* by the hospital laboratory. Incubation of the bacteria in the chip containing antibiotics, for 2 h (Figure 6) or 4 h (Supplemental Files; Figure S9), demonstrated resistance to ampicillin and gentamicin, but susceptibility to amikacin. These results were in correlation with the results obtained by the hospital laboratory. 

### 3.5. Application of Machine Learning for Classification of Microscopic Images of Bacterial Cells for AST

**Figure 7:**
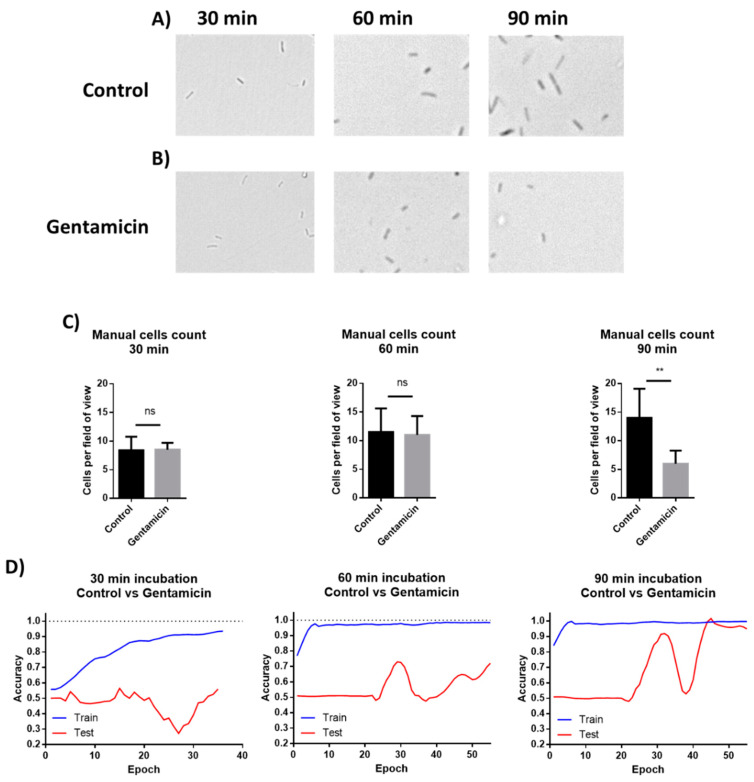
Manual cells counting and deep learning for AST. (**A**) *E. coli* cells were incubated without antibiotics for 30, 60, and 90 min. (**B**) Cells were incubated in wells with drop-dried antibiotic for 30, 60, and 90 min. (**C**) Manual counting of the cells after 30, 60 and 90 min of incubation. n = 8. Error bars indicate standard deviation. (**D**) Machine learning plots, as tested after 30, 60 and 90 min of incubation. ** *P* ≤ 0.01.

**Figure 8:**
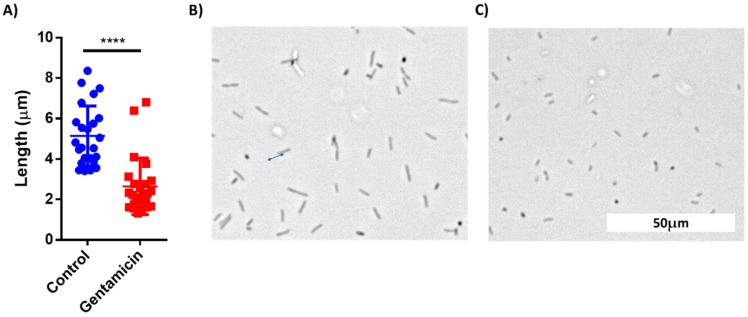
Comparison of cell length of treated and untreated E. coli cells after 60 min of incubation. (**A**) Cell length distribution between the control group and gentamicin-treated group. (**B**) A Brightfield microscopy photo of control cells. (**C**) A photo of gentamicin-treated cells. n = 25. Error bars indicate standard deviation (SD). **** *P* < 0.001.

The images from the AST discussed above were manually analyzed. However, deep learning models can be applied to process visual data. To demonstrate the possibility of using machine learning (ML) for AST analysis, *E. coli* cells were incubated in LB inside a 48-well plate containing drop-dried gentamicin. The wells were photographed 30, 60 and 90 min post-seeding (Figure 7A,B). The images were used to train a residual neural network (ResNet). *E. coli* cells were also counted manually at the same time points. The manual count found no significant differences in cell numbers after 30 min and after 60 min while it did record a significant difference after 90 min of incubation (Figure 7C). The ML model found no differences in cell counts after 30 min, but significantly improved after 60 min, yielding 70% accuracy. The accuracy increased to 98% after an additional 30 min of incubation (Figure 7D). These results demonstrate that ML can both provide accurate classification after 90 min of incubation, a time point at which differences in cell numbers were visibly detectable, and also detect some other changes that are not based on cell number at least 30 min before the differences in cell numbers became visible. One of the observed changes is the cells’ length (Figure 8).

## 4. Conclusions

In summary, this work presented a proof-of-concept study of a device for rapid UTI diagnostic and AST. For the UTI diagnostics, a single-channel microfluidic chip capable of detecting 5 × 10^3^ cells/mL, a clinically relevant concentration of bacterial cells in urine, within 10 min, was developed. The same device can also be used for AST when equipped with a 4- or 16-channel chip. Application of both hydrophilic and superhydrophobic coatings to the chip’s surface enabled separation between the test zone and the sample-receiving zone as well as between the channels themselves. Rapid (2 h), in-chip AST of *E. coli*, as well of *K. pneumoniae* that was isolated from urine of a UTI patient, was demonstrated. Using a well plate, it was demonstrated that deep learning could accelerate the process, providing results within 60 min, with an accuracy of 70%. After 90 min of incubation, the accuracy increases to 98%. The presented prototypes can be further developed as a platform for efficient and rapid UTI diagnostics and AST.

## Supplementary Materials

The following are available online at https://www.mdpi.com/article/10.3390/s21175902/s1, Video S1: Silicon glue deposition on a glass cover. Video S2: Fully assembled Chip #1 attached to a 15 mL tube. Videos S3 and S6: Demonstration of the spontaneous flow-back of liquid and channel separation in a chip with hydrophilic and hydrophobic coatings. Videos S4 and S5: Demonstration of absence of spontaneous flow-back of liquid in a chip without hydrophilic and hydrophobic coatings. Figure S1: Chip assembling process. Figure S2: An EVOS microscope adaptor to hold the chip. Figure S3: Demonstration of lack of spontaneous liquid flow into the channels of the chip. Figure S4: Flow between two channels when no superhydrophobic coating is applied. Figure S5: Demonstration of titanium dioxide-based superhydrophobic coating. Figure S6: The green dye does not diffuse out of the microchannel. Figure S7: A custom-made adapter for a standard table centrifuge. Figure S8: Brightfield microscopy photos of E. coli cells incubated for 4 h inside microchannels in a chip with or without addition of antibiotics. Figure S9: *Klebsiella pneumoniae* isolated from a clinical urine sample incubated for 4 h in chip with or without addition of antibiotics.

## Abbreviations

**Abbreviations**	**Full Name**
UTIs	Urinary tract infections
AST	Antibiotic susceptibility test
*K. pneumonia*	*Klebsiella pneumonia*
*E. coli*	*Escherichia coli*
CDC	Disease Control and Prevention
GDP	Gross domestic product
CFUs	Colony-forming units
POC	Point-of-care
FOTS	1H,1H,2H,2H-perfluorooctyltriethoxysilane
FDA	Food and Drug Administration
LB	Lysogeny broth
ResNet	Residual neural network
ML	Machine Learning
