# Supplementary material for: BactoSpin: Novel Technology for Rapid Bacteria Detection and Antibiotic Susceptibility Testing
Source: Sensors (Basel). 2021 Sep 2;21(17):5902. doi: 10.3390/s21175902 (PMC8434515; doi:10.3390/s21175902)
Supplement: Supplementary file 1 [file sensors-21-05902-s001.zip › sensors-1353088-supplementary.pdf]

BactoSpin: novel technology for rapid bacteria detection and antibiotic susceptibility testing

Vlad Shumeiko[1], Guy Hidas[2], Chen Nowogrodski[1], Yariv Pinto[3], Ofer Gofrit[2], Mordechai Duvdevani[2], Oded Shoseyov[1*].

[1] Faculty of Agriculture, Food and Environment, The Hebrew University of Jerusalem, Rehovot 7610001, Israel

[2] Department of Urology, Hadassah Medical Center, Faculty of Medicine, Hebrew University of Jerusalem, Jerusalem 9112001, Israel.

[3] Center for Functional and 3D Printing, Center for Nanoscience and Nanotechnology, The Hebrew University of Jerusalem, Jerusalem 9190401, Israel

* Correspondence: shoseyov@agri.huji.ac.il

Supplemental materials

List of supplemental videos:

- **Video S1**: Silicon glue deposition on a glass cover using a syringe and Hyrel3D printer.
- **Video S2**: Fully assembled chip #1 attached to a 15 ml tube holding 10 ml of colored water. The liquid easily enters and exits the chip – thus enabling flow-back of sample after centrifugation.
- **Video S3 and Video S6**: Demonstration of the spontaneous flow-back of liquid and channel separation in a chip with hydrophilic and hydrophobic coatings.
- **Video S4 and Video S5**: Demonstration of absence of spontaneous flow-back of liquid in a chip without hydrophilic and hydrophobic coatings.

Supplemental figures:

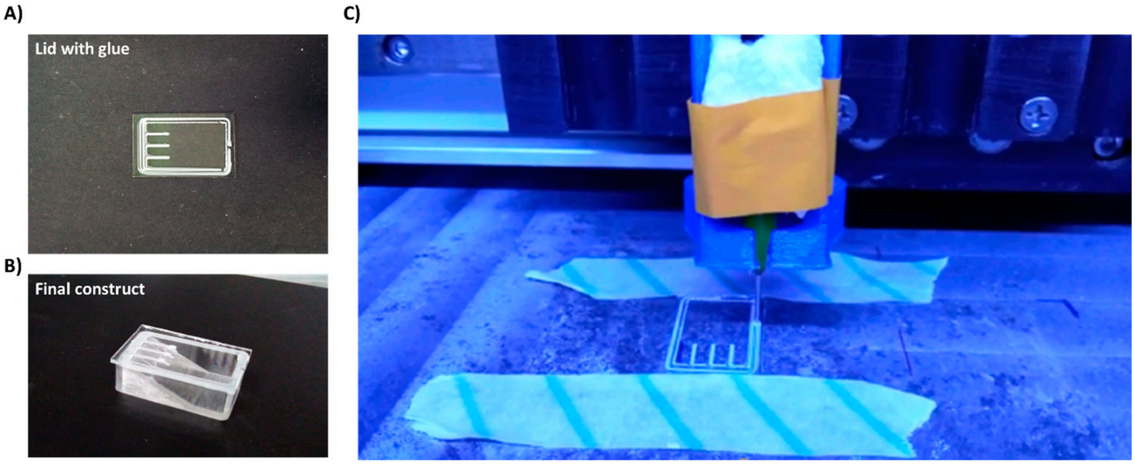

Figure S1: Chip assembling process. A) A glass cover (cap) of the chip with silicon glue on it. B) An assembled chip with all coatings and with the cap. C) A photo of the glue deposition process using a Hyrel3D printer.

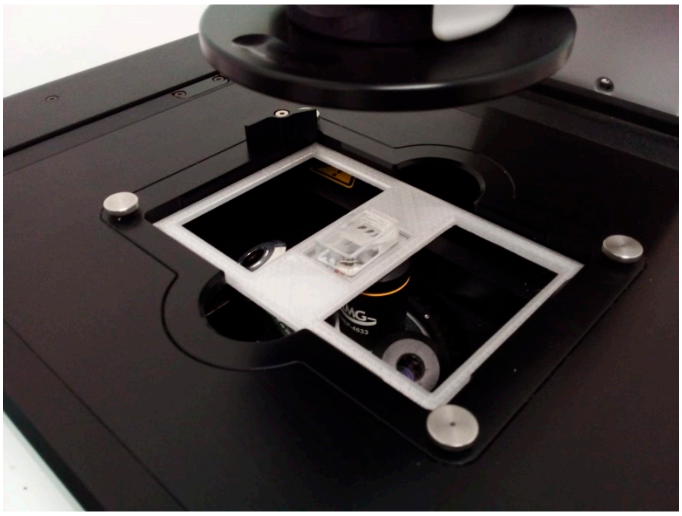

Figure S2: An EVOS microscope adaptor to hold the chip.

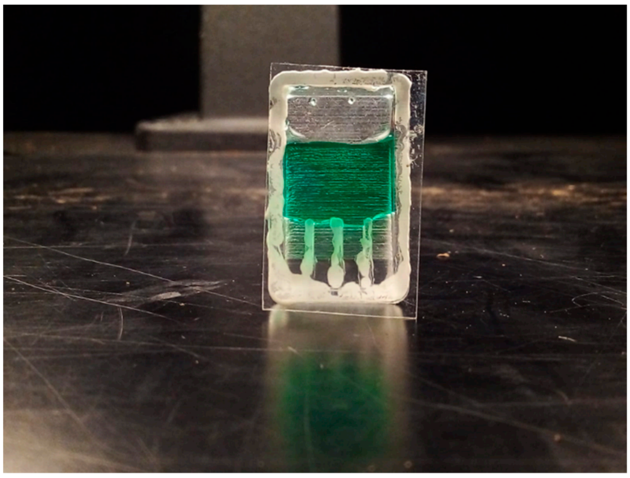

Figure S3: Demonstration of lack of spontaneous liquid flow into the channels of the chip coated with superhydrophobic and hydrophilic coatings without centrifugation.

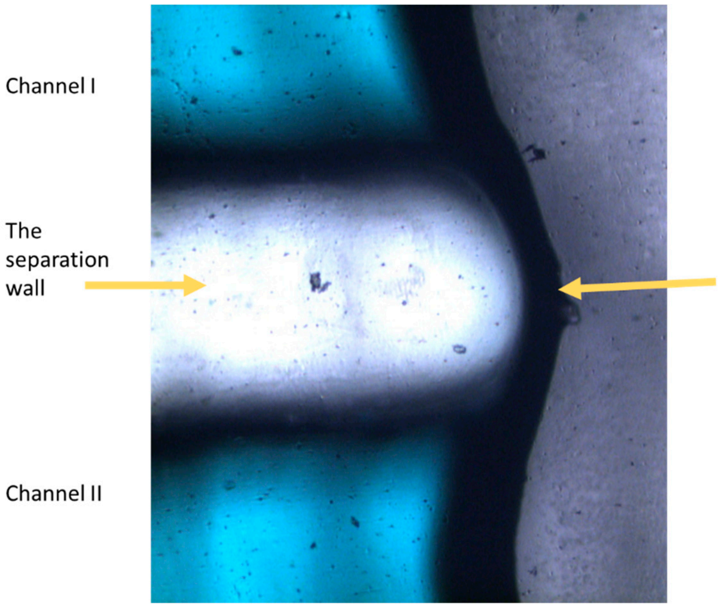

Figure S4: Flow between two channels when no superhydrophobic coating is applied.

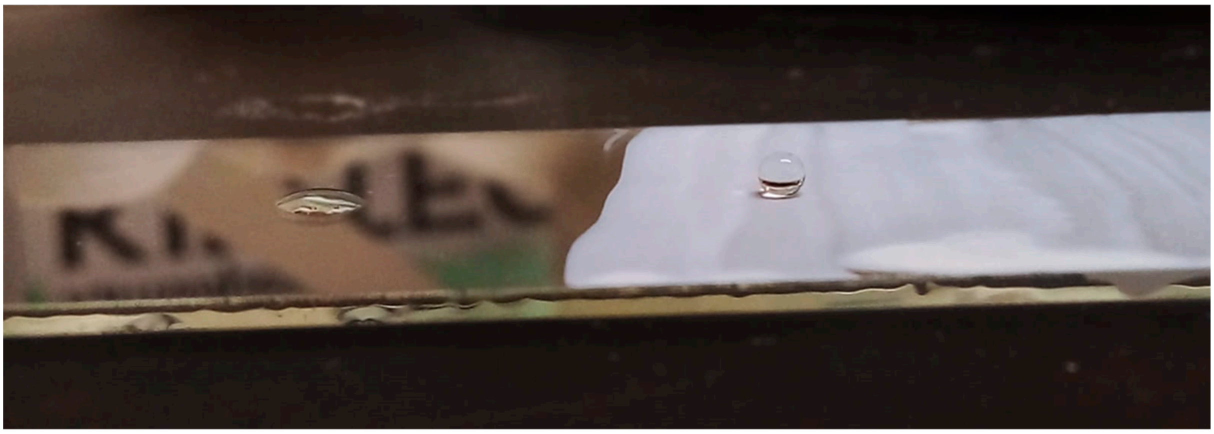

Figure S5: Demonstration of titanium dioxide-based superhydrophobic coating of a glass slide with no coating on the left side and coating on the right side. A water drop shows a very low contact angle with the uncoated glass, versus a very high contact angle on the coated side.

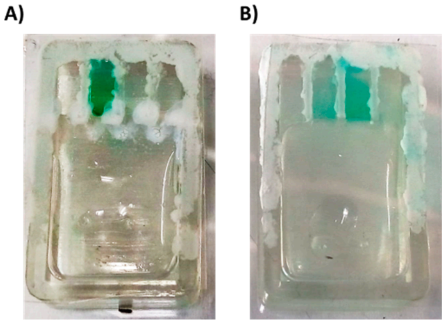

Figure S6: The green dye does not diffuse out of the microchannel after 4 hours of incubation in a chip with a superhydrophobic coating, (A) while it does diffuse when no coating is applied (B).

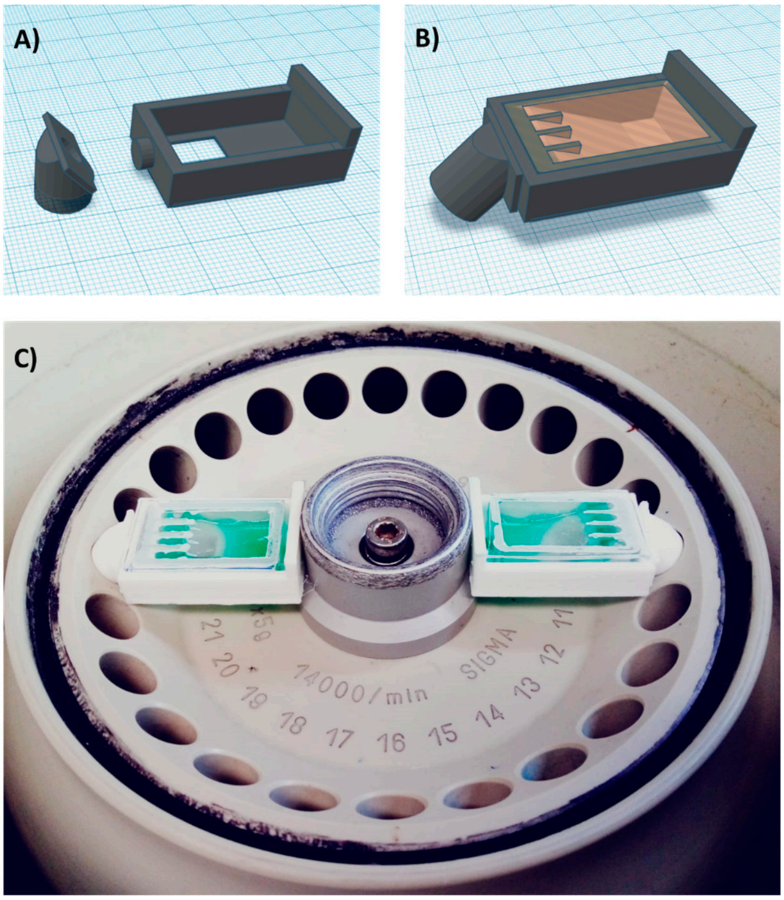

Figure S7: A custom-made adapter for a standard table centrifuge. A) A 3D model of the adapter. B) A 3D model of the adapter holding the chip. C) A photo of the printed adapter carrying an assembled chip.

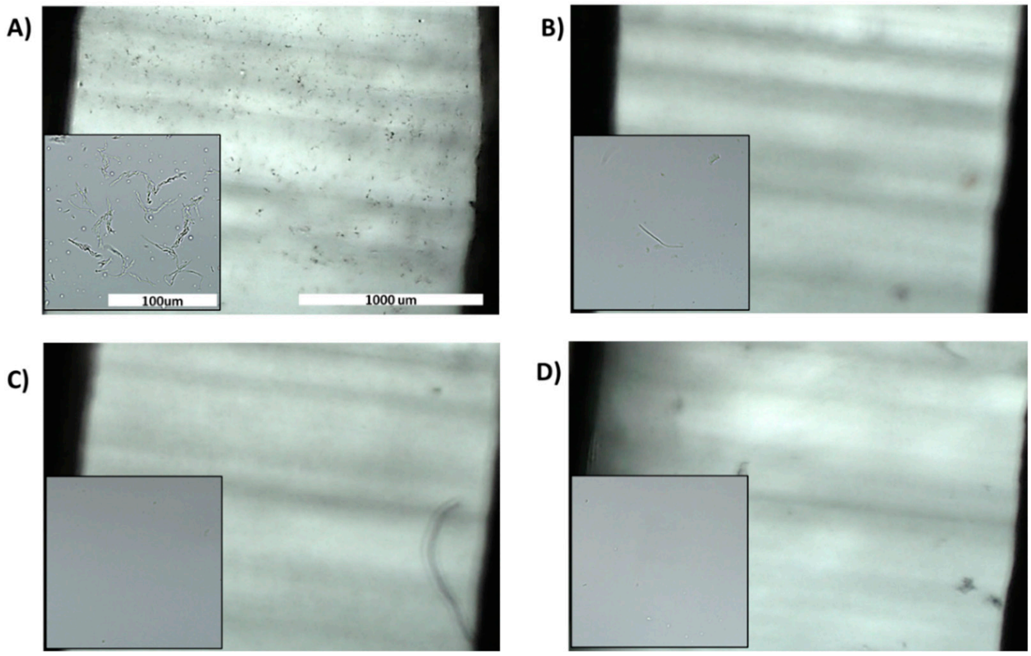

Figure S8: Brightfield microscopy photos of E.coli cells incubated for 4 hours inside microchannels in a chip with a superhydrophobic and hydrophilic coatings. A) The control channel as observed with 4x objective. The insert photo was taken using a 40x objective. A channel that contains B) ampicillin,. C) gentamicin, D) amikacin.

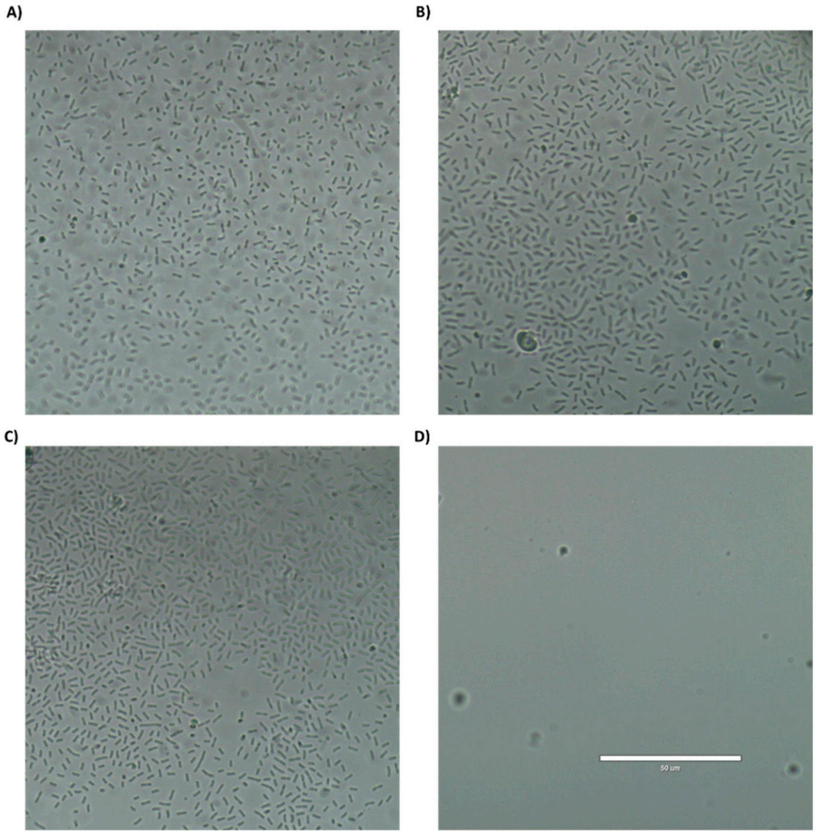

Figure S9: Klebsiella pneumoniae isolated from a clinical urine sample were incubated for 4 hours in the chip with superhydrophobic and hydrophilic coatings. A) Control channel. Channel with drop-dried B) ampicillin, C) gentamicin, D) amikacin.
